# Uptake and depuration of gold nanoparticles in *Daphnia magna*

**DOI:** 10.1007/s10646-014-1259-x

**Published:** 2014-05-27

**Authors:** L. M. Skjolding, K. Kern, R. Hjorth, N. Hartmann, S. Overgaard, G. Ma, J. G. C. Veinot, A. Baun

**Affiliations:** 1 Department of Environmental Engineering, Technical University of Denmark, Miljøvej, B113, 2800 Kgs. Lyngby, Denmark; 2 Department of Chemistry, University of Alberta, Edmonton, AB T6G 2G2 Canada; 3 NRC-National Institute for Nanotechnology, Edmonton, AB T6G 2M9 Canada

**Keywords:** Kinetics, Feeding, Size, Test design, Au

## Abstract

This study presents a series of short-term studies (total duration 48 h) of uptake and depuration of engineered nanoparticles (ENP) in neonate *Daphnia magna*. Gold nanoparticles (Au NP) were used to study the influence of size, stabilizing agent and feeding on uptake and depuration kinetics and animal body burdens. 10 and 30 nm Au NP with different stabilizing agents [citrate (CIT) and mercaptoundecanoic acid (MUDA)] were tested in concentrations around 0.5 mg Au/L. Fast initial uptake was observed for all studied Au NP, with CIT stabilized Au NP showing similar rates independent of size and MUDA showing increased uptake for the smaller Au NP (MUDA 10 nm > CIT 10 nm, 30 nm > MUDA 30 nm). However, upon transfer to clean media no clear trend on depuration rates was found in terms of stabilizing agent or size. Independent of stabilizing agent, 10 nm Au NP resulted in higher residual whole-animal body burdens after 24 h depuration than 30 nm Au NP with residual body burdens about one order of magnitude higher of animals exposed to 10 nm Au NP. The presence of food (*P. subcapitata*) did not significantly affect the body burden after 24 h of exposure, but depuration was increased. While food addition is not necessary to ensure *D. magna* survival in the presented short-term test design, the influence of food on uptake and depuration kinetics is essential to consider in long term studies of ENP where food addition is necessary. This study demonstrates the feasibility of a short-term test design to assess the uptake and depuration of ENP in *D. magna*. The findings underlines that the assumptions behind the traditional way of quantifying bioconcentration are not fulfilled when ENPs are studied.

**Electronic supplementary material:**

The online version of this article (doi:10.1007/s10646-014-1259-x) contains supplementary material, which is available to authorized users.

## Introduction

An extensive literature review of all papers on environmental effects of engineered nanomaterials and nanoparticles (ENM and ENP, respectively) published before 2009 were published in 2010 concluding that, “only a few studies have dealt with bioaccumulation of metal nanoparticles” (Stone et al. 2010). The main focus in the scientific literature dealing with environmental effects of ENM has been on toxicity aspects and to a much lesser extends on uptake and depuration of ENM. Since 2009 the literature on uptake and depuration of ENM has been expanding (>50 studies on terrestrial and aquatic species are available at present) but comparisons and generalizations are difficult due to the large variety of ENM tested, lack of standardized test procedures and differences between test organisms. A review on test methods and test organisms by Handy et al. (2012a) underlined the need for modification of ecotoxicity and environmental fate test methods to ENM in terms of e.g. test species, test media and concentrations monitoring during test. Especially, for chronic studies which can last for weeks (e.g. 21 days using the OECD 211 Reproduction test with *Daphnia magna* (OECD 2008)) the afore mentioned parameters becomes critical as the tests often become more complex and include even more complicating factors such a semi-static exposure conditions and feeding of the animals.

As outlined by Handy et al. (2012a) the choice of organism is of crucial importance, and with respect to uptake of ENM *D. magna* is considered to be a relevant test organism due to feeding traits, general behavioural habits and placement in the food chain (Baun et al. 2008a, b). *D. magna* filters water to catch particles (mainly algae) in the size range 0.4–40 μm (Gophen and Geller 1984; Geller and Muller 1981). Different agglomeration patterns of Au NP are observed for different stabilizing agents thus actively affecting the size of ENM in water (Liu et al. 2012). Due to agglomeration of ENM in freshwater it is therefore likely that ENM agglomerates will be ingested. This has been demonstrated in a number of studies with Daphnia spp. and different types of ENM or agglomerates e.g. Lovern et al. (2008), Baun et al. (2008a, b),, Petersen et al. (2009), Zhu et al. (2010), Croteau et al. (2011), Hartmann et al. (2012) and Hu et al. (2012). As a part of the digestion process *Daphnia* spp. are known to take in water (Gillis et al. 2005) thus small particles can directly be taken up from the water column (Rosenkranz et al. 2009). Also attachment to algae is a possible route of uptake for ENM and ENP agglomerates. Uptake across the gut section generally requires transport across a biological membrane. This transport is largely controlled by passive diffusion, active uptake, transport through ion channel or carrier mediated transport (Sijm et al., 2007). However, for ENP different types of cytosis could be the mechanism of uptake. For metal-based ENP susceptible to release metal ions a combination of well-understood mechanisms could be used to describe the uptake both through phagocytosis and ion theory. Silver NP studied by Zhao and Wang (2010) showed uptake rates being biphasic with difference for high and low concentration. Higher uptake rates at higher concentrations were assumed to be due to particle ingestion. However, uptake at lower concentrations could be well described by first-order uptake kinetics (Zhao and Wang 2010). Histological studies by e.g. Lovern et al. 2008 showed Au NP in the gut section. Similarly, Au NP were found solely in the gut section of the filter-feeding bivalve (*Corbicula fluminea*) after exposure to CIT coated Au NP (Hull et al. 2011). For the lugworm (*Arenicola marina*) exposed to TiO_2_ NP with agglomeration size of >200 nm, no uptake past the gut lumen was observed (Galloway et al. 2010). Conversely, nano-sized polystyrene beads (20 nm) were found in the oil droplets of *D. magna* (Rosenkranz et al. 2009). Other studies have also found different uptake behaviour due to size and shape as shown for different shaped nanocrystals of Cu_2_O NP in *D. magna* (Fan et al. 2011), for different sized CuO in deposit-feeding snails (*Potamopyrgus antipodarum*) (Pang et al. 2012), and Au NP of different sizes in tellinid clams (*Scrobicularia plana*) (Pan et al. 2012).

From the above studies the size, shape and stabilizing agents or coatings have been identified as factors that may affect the potential uptake and depuration of ENP. Therefore, this study aims to investigate the particle specific uptake of engineering nanoparticles as a function of particle size and stabilizing agent and evaluate the proposed test design in terms of test duration and mass balances of the added ENP in the test setup. Furthermore, it was studied if feeding has an influence on the uptake and depuration behaviour of Au NP. Throughout this study the term uptake is used to describe particles entering the test organism and does not necessarily imply that translocation or membrane passage occurred. The study was carried out using the invertebrate *D. magna* as model organism and Au NP with two stabilizing agents and two sizes. Gold was chosen as a study particle for a number of reasons: (I) Au NP exhibit a low toxicity thus minimizing toxicity effects interfering with uptake and depuration kinetics, (II) Even at the nano-scale gold is a rather inert material and in water minimal dissolution will occur, (III) Through a well-controlled synthesis, Au NP with different sizes and functionalizations can be produced, (IV) There is a low background concentration of gold in the aquatic environment and (V) Low detection limit both chemically and by transmission electron microscopy (TEM) (Alkilany and Murphy 2010; Mermet 2005). Furthermore, Au NP is on the OECDs “List of Representative Manufactured Nanomaterials”, which is a list of thirteen NM that is about to enter, or already have entered into commerce (OECD 2010).

## Materials and methods

### Test organism

The *D. magna* culture originates from Birkedammen, Denmark in 1978 and has since then continuously been cultured at the Department of Environmental Engineering, Technical University of Denmark. For culturing, 12 adult animals were kept in a 1 L glass beaker filled with 800 mL Elendt M7 medium (OECD 2004). The culture medium was renewed twice a week, and the animals were fed with green algae (*P. subcapitata*) three times a day for 15 min via pump. The culture was maintained in a temperature-controlled room at 20 ± 1 °C with a 16:8 h light–dark cycle.

### Chemicals

Four different Au NP suspensions were obtained from the University of Alberta, Canada. Nanoparticles with a particle size of 10 and 30 nm were stabilized with CIT or MUDA, respectively. CIT stabilized Au NP were prepared in aqueous media by heating a solution of HAuCl_4_–2H_2_O (0.25 mM, 3.75 mM tribasic salt, 1 L) to 90 °C. The solution was heated for 1 h over which time its colour gradually changed to grey and finally purple/red. The CIT Au NP solutions were subsequently purified by dialysis. Dialysis was done on 1000 mL of stock solution which was divided into two 500 mL fractions and placed in Lot Number 3244650 dialysis tubing (approximate molecular weight cut off = 8,000 Daltons). The filled tubes were submerged in distilled water for 4 days and the bath water was changed at 12 h intervals. MUDA stabilized Au NP were prepared by addition of 500 mL fraction of 30 nm CIT capped Au NP stock solution directly to an ethanol solution of 11-MUDA (0.12 g, 3 mL). The mixture was stirred in subdued light for one week. The resulting solution was then purified by dialysis using the procedure outlined above.

*Aqua regia* (nitrohydrochloric acid) was prepared by mixing analytical grade HNO_3_ and HCl (Sigma-Aldrich) at a ratio of 1:3 (v/v).

### Preparation of Au NP test solution

The test dilutions for the toxicity, uptake and depuration studies were prepared immediately prior to use by adding the required amount of stock solution to a volumetric flask containing Elendt M7 medium (OECD 2004). The flask was hereafter filled up to the mark with Elendt M7 medium. No stirring or ultra-sonication was applied.

### Characterization with transmission electron microscopy and dynamic light scattering

Stock solutions were characterized in MilliQ water by placing a drop on copper grids (Cu, 3 mm, 250 mesh square, SPI-grids) and letting it dry for 1 h before analysing it with TEM (Valeta CM 100 Phillips, operating voltage 100 kV). FT-IR spectroscopy was performed on powder samples using a Nicolet Magna 750 IR spectrophotometer. X-ray photoelectron spectroscopy (XPS) was acquired in energy spectrum mode at 210 W, using a Kratos Axis Ultra X-ray photoelectron spectrometer. Samples were prepared as films drop-cast from solution onto a copper foil substrate.

Size of Au NP in Elendt M7 was determined by Dynamic Light Scattering using a Zetasizer Nano-ZS at 20 °C. A backscattering angle of 173° was used to determine the observed light. Each agglomeration experiment was run with three replicates using 30 measurement runs of 1 mL sample solution in 1 × 1 cm plastic cuvettes. Stokes–Einstein equation was used to calculate the hydrodynamic diameter of the Au NP using the cumulant method for fitting the autocorrelation function (Kretzschmar et al. 1998).

### Acute toxicity test

A series of acute toxicity studies were carried out to determine appropriate concentrations to be used in uptake and depuration studies. All acute toxicity tests were carried out following the OECD 202 guideline for acute immobilization tests with *Daphnia* sp. (OECD 2004). *D. magna* neonates (<24 h old) were used for testing. The tested concentrations ranged from 0.1 to 10 mg/L and the number of immobile animals was counted after 24 and 48 h. Toxicity of the reference compound (potassium dichromate), pH-values, and oxygen concentrations were within the validity criteria specified by the guideline (OECD 2004) (Table S1).

### Uptake and depuration experiments

Uptake and depuration experiments, including a 24 h uptake period followed by a 24 h depuration period were carried out in suspensions of 0.5 mg Au/L with the differently sized and capped Au NP (10 and 30 nm with both CIT and MUDA as stabilizing agent). 5–10 *D. magna* neonates were placed into a 100 mL glass beaker containing 25 mL of Au NP suspension. Furthermore, three control beakers without addition of Au NP were included. Beakers were incubated at 20 °C in the dark and mortality was noted for each beaker at the end of the test. *D. magna* were sampled after 1, 2, 4, 6 and 24 h by sacrificing the mobile animals of three beakers at each sampling time. Immobile *D. magna* was not used for the chemical analysis. Immediately after sampling the animals were rinsed in a 10 % diluted *aqua regia* for approximately 30 s after which they were stored in 20 mL glass vessels for chemical analysis. At the end of the 24 h exposure period all mobile animals in the remaining beakers were transferred to fresh Elendt M7 medium for the depuration study. Here the animals from three beakers were sampled at 1, 2, 4, 6, and 24 h after transfer to clean media. All sampled *D. magna* were stored in the dark at room temperature up to the chemical analysis. In addition to the above described tests, animals from three beakers were sacrificed daily (at 48 and 72 h) in a preliminary prolonged study of depuration. To estimate the weight of *D. magna* a parallel test setup scaled to approximately 100 *D. magna* neonates were carried out using same test conditions as described above. At the end of the test period (24 h) the *D. magna* were transferred to an oven dried G55 filter and dried in oven at 105 °C for 24 h before weighing.

### The influence of feeding during uptake and depuration of Au NP

For the studies of the influence of feeding on uptake and depuration in *D. magna*, ten neonates were placed in 100 mL beakers containing 25 mL Elendt M7 medium with a concentration of 0.4 mg Au/L (10 nm CIT Au NP). An additional three controls containing clean Elendt M7 medium were prepared for sampling at the end of the tests (48 h). Test beakers were incubated in the dark at 20 ± 1 °C for the duration of the test and three beakers were sampled per time i.e. 30 animals. Sampling for ENP uptake was done at 1, 2, 4, 8 and 24 h. At end of the uptake period all *D. magna* in the remaining beakers were transferred to beakers with 25 mL clean Elendt M7 media after a quick rinsing step (also in Elendt M7 media) to remove Au NP from exoskeleton. Sampling in triplicates for depuration was done at 25, 26, 28, 32, and 48 h after test start. For sampling, *D. magna* were transferred from the test beaker to a nylon filter with a plastic pipette and rinsed in a 10 % dilution of *aqua regia* for 30 s prior to storage in 20 mL glass vials. The feeding experiments were carried out for four different scenarios: with or without food for the uptake and depuration. Food (*P. subcapitata*, 0.2 mg C/animal/day, corresponding to 2 × 10^7^ cells/mL measured with Z2 Coulter Counter, Beckman Coulter™) was administered either at the beginning of the exposure period and/or at the beginning of the depuration period. This experiment without feeding is considered the base line study to which identical studies with addition of food during uptake and/or depuration is compared. To estimate the weight of *D. magna* a parallel test setup (with and without food) scaled to approximately 100 *D. magna* neonates were carried out using same test conditions as described above. At the end of the test period (24 h) the *D. magna* were transferred to an oven dried G55 filter and dried in oven at 105 °C for 24 h before weighing.

### Mass balance of Au after exposure

Mass balances were determined for the test system using 30 nm CIT Au NP and 30 nm Au NP from National Institute of Standards and Technology (NIST). The latter were used as a reference material for recovery in the test system as well as for acid digestions and analytical determination of gold. In the mass balance experiments five neonates (<24 h) were put into 100 mL glass beakers filled with 25 mL of 0.5 mg/L Au NP suspension in Elendt M7 medium. After 24 h exposure period, animals were removed with a fine nylon mesh. Subsequently, all animals from one beaker were put simultaneously into 20 mL of diluted *aqua regia* (ratio 1:10) for approximately 30 s. Hereafter, they were again transferred with a plastic pipette onto nylon net. The net was dried from the bottom with a paper towel to remove excess liquid and the animals were transferred with the help of a metal tip into a glass vial. The glass vial was weighted before adding 2 mL of *aqua regia*. All vials were stored in the dark at room temperature for at least 24 h, before they were weighted again. Prior to chemical analysis 8 mL of distilled water were added. To test for Au adsorbed to the glassware, beakers used for the experiments were rinsed twice with 1 mL of *aqua regia* and hereafter two times with 4 mL of distilled water. The 10 mL were transferred quantitatively to a 20 mL glass vial and stored in the dark at room temperature until chemical analysis. To test for Au in the solution 5 mL of the test dilutions was taken to determine the initial concentration.

### The influence of sorption during uptake of Au NP

An experiment were conducted with animals incapable of actively consuming particles in order to determine the role of sorption to the animals in the interpretation of body burdens found in uptake and depuration studies. For this the uptake and depuration test setup (see section *Uptake and depuration experiments*) was used with *D. magna* that were put to death in a 16.9 % ethanol solution in Elendt M7 medium immediately before the beginning of the tests. Life signs were checked visually in a microscope to ensure that no movement was present. Immediately hereafter the *D. magna* were rinsed in a clean Elendt M7 medium and transferred to the test beakers, where they sank to the bottom of the solution.

### Chemical analysis

Prior to chemical analysis all samples were digested in *aqua regia* at room temperature for at least 24 h in the dark. During the digestion procedure no heat or other additional treatment was applied. Before the chemical analysis distilled water was added and the samples were decanted into disposable plastic vials. Chemical analysis was carried out with ICP-OES (Varian Vista-MPX CCD simultaneous ICP-OES) using the following settings: max standard error ±15 %, scanning with internal standard Y-377.433. Gold standards used: Au-208.207, Au-211.068, Au-242.794, Au-267.594.

### Data treatment

For the analysis of acute toxicity data the program ToxCalc™ v5.0 was used. The method used in this study was the point estimate method which is linear regression by maximum likelihood estimation where the probit model is used (Tidepool Scientific). For the quantification of data from the uptake and depuration studies, rates for the initial uptake (k_1,initial_) and depuration (k_2,initial_) were modelled using first-order rate model given in Eq. 1 using non-linear curve fitting (GraphPad Prism v5.0).

1 $$ C_{t} = \frac{{C_{w} k_{u} }}{{k_{e} }}\left( {1 - e^{{ - k_{e} t}} } \right) $$

where *C*_*t*_ is the concentration in the organism at time *t*, *C*_*w*_ is the water phase concentration, *k*_*u*_ is the uptake rate and *k*_*e*_ is the elimination rate. To accommodate for changing water concentration the initial water phase concentration was used to estimate a low uptake rate (Start) and the final water phase concentration was used to estimate a high uptake rate (Final).

All experiments were carried out in triplicates and for each data set the mean and standard deviation (SD) was calculated. Mean values were recorded as mean ± 1 SD throughout this paper. For comparisons of two groups the Kruskal–Wallis test and Dunn’s multiple comparison test was used and data was considered statistically significant different at p value < 0.05 (GraphPad Prism v5.0).

## Results

### Characterization and stability of Au NP

**Fig. 1:**
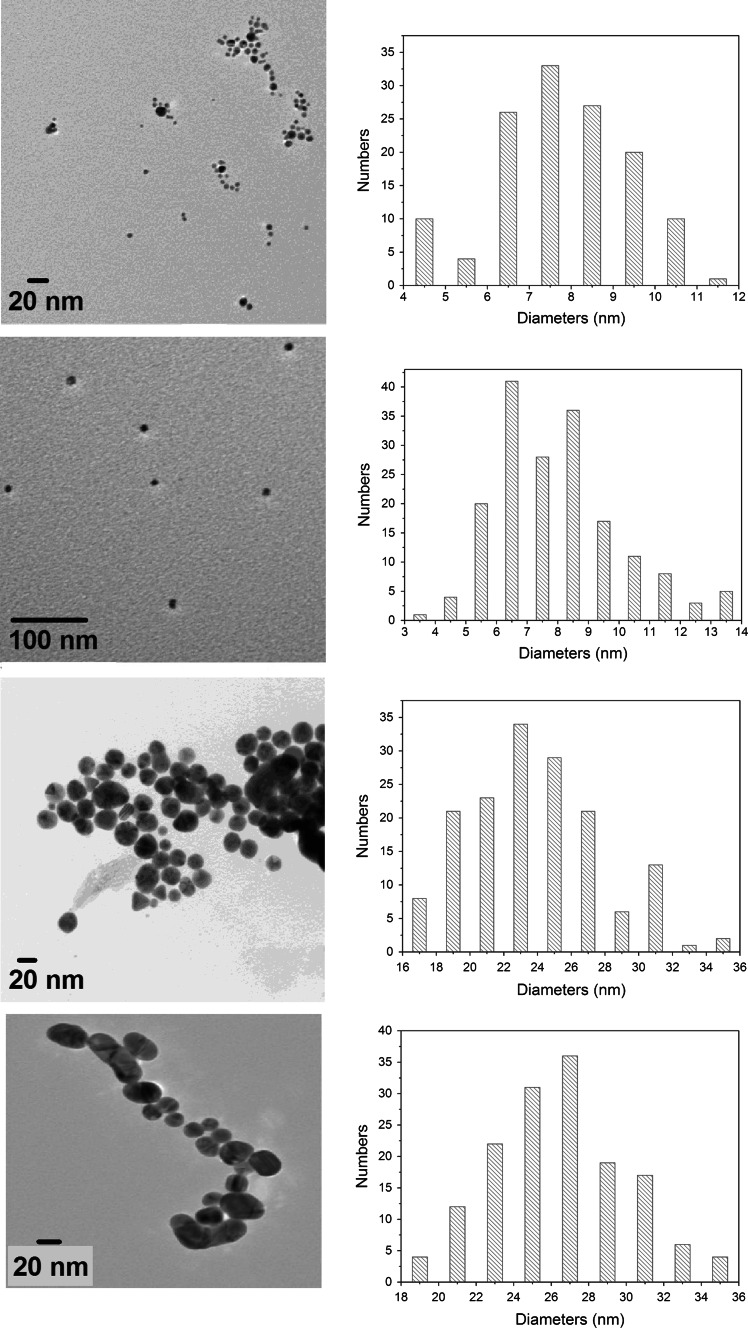
TEM images and statistical size distribtution of Au NP in MilliQ water from top: CIT 10 nm Au NP (d = 7.5 ± 3 nm), MUDA 10 nm Au NP (d = 8.0 ± 3 nm), CIT 30 nm Au NP (d = 23.0 ± 9 nm) and MUDA 30 nm Au NP (d = 27.0 ± 6 nm). *MUDA* mercaptoundecanoic acid, *CIT* citrate

From the TEM pictures of Au NP dispersed in MilliQ water it is seen that the particles’ shapes and sizes corresponds to the suppliers information and was generally found to be homogenous throughout the samples (Fig. 1). IR spectra and XPS after ligand exchange in aqueous solution showed no peaks of non reacted Au ions (Fig. S1). Initial measurements (0 h) using DLS to determine the size distribution in Elendt M7 media showed bimodal volume distributions for the MUDA 10 nm Au NP with two distinct peaks with 71 % in the range of 20 ± 5 nm and a 2nd peak of 28 % in the range of 142 ± 53 nm. MUDA 30 Au NP showed a similar trend in volume distribution with 82 % in the range of 109 ± 42 nm and 18 % in the range 23 ± 5 nm. CIT 10 nm Au NP showed 91 % in the range of 14 ± 4 nm and 9 % in the range of 112 ± 47 nm. CIT 30 nm Au NP showed an increase in size to 225 ± 61 nm. After 24 h all Au NP except CIT 10 nm was found in the 1st peak (Table 1). Agglomeration to larger sizes was observed for all tested Au NP after 24 h. The zeta-potential of the Au NP after 24 h in Elendt M7 medium was found to be 13 ± 6 mV, 14 ± 6 mV, 16 ± 6 mV and 16 ± 5 mV for MUDA 10 nm, CIT 10 nm, CIT 30 nm and MUDA 30 nm respectively. All Au NP particles were found to have an incipient stability (±10 to ±30 mV) in Elendt M7 media within the time frame used for uptake tests (24 h).

**Table 1:** Size peaks recorded (percentage of particles in this range) and zeta-potential of Au NP in Elendt M7 after 0 and 24 h measured by dynamic light scattering and transformation to volume-based distribution (mean ± standard deviation; n = 3)

Test compound	Size peak 1 (nm)		Size peak 2 (nm)		Zeta-potential (nm)	
	t = 0	t = 24 h	t = 0	t = 24 h	t = 0	t = 24 h
MUDA^a^ 10 nm Au NP	20 ± 5 (71 %)	229 ± 60 (100 %)	142 ± 53 (29 %)	N/A	−14 ± 7	−16 ± 5
MUDA^a^ 30 nm Au NP	109 ± 42 (82 %)	279 ± 53 (100 %)	23 ± 5 (18 %)	N/A	−15 ± 9	−13 ± 6
Citrate 10 nm Au NP	14 ± 4 (91 %)	188 ± 48 (60 %)	112 ± 47 (9 %)	20 ± 4 (40 %)	−14 ± 8	−14 ± 6
Citrate 30 nm Au NP	225 ± 61 (100 %)	328 ± 61 (100 %)	N/A	N/A	−14 ± 9	−16 ± 6

*N/A* No applicable data

^a^Mercaptoundecanoic acid

### Mass balance of Au in the test system

No sorption of Au NP to the exterior surfaces of *D. magna* was observed in the study with dead animals as all analysed samples had a gold content below the detection limit of the ICP-OES (1.34 ± 0.06 μg/L). From a series of preliminary studies it was found that rinsing exposed animals with diluted *aqua regia* upon transfer to depuration beakers was superior to distilled water in terms of recovery (data not shown). The results from the mass balance tests showed a recovery of 104 ± 6.5 % (n = 3) after the 24 h incubation period compared to the measured initial amount of gold added to the test system. The amount of gold recovered was divided between the following four fractions: 0.30 ± 0.24 % in the *aqua regia* used for rinsing the exterior of the animals, 38 ± 2.4 % in the acid digested animals, 32 ± 2.9 % adsorbed to the glass of the test vessel and 30 ± 4.7 % in the water phase.

### Acute toxicity testing of Au NP

The results from the acute toxicity tests are shown in Table 2. It is seen that MUDA Au NP was generally more toxic than the CIT Au NP. From the values presented in Table 2 sub-lethal exposure concentration of 0.5 mg Au/L was used based on the acute toxicity of the MUDA Au NP as they showed the highest toxicity of the tested Au NP (Table 2).

**Table 2:** Results from 24-h *D. magna* acute toxicity test with Au NP with different stabilizing agents. Effect concentrations and corresponding 95 % confidence intervals are all in mg/L

Test compound	EC10, 24 h (mg Au/L)	EC10, 48 h (mg Au/L)
MUDA^a^ 10 nm Au NP	0.73 (0.07; 2.4)	0.14 (0.05; 0.25)
MUDA^a^30 nm Au NP	2.1 (0.49;5.6)	0.14 (0.0005;0.45)
Citrate 30 nm Au NP	>10	>10

^a^Mercaptoundecanoic acid

### Uptake and depuration of Au NP in D. magna

**Fig. 2:**
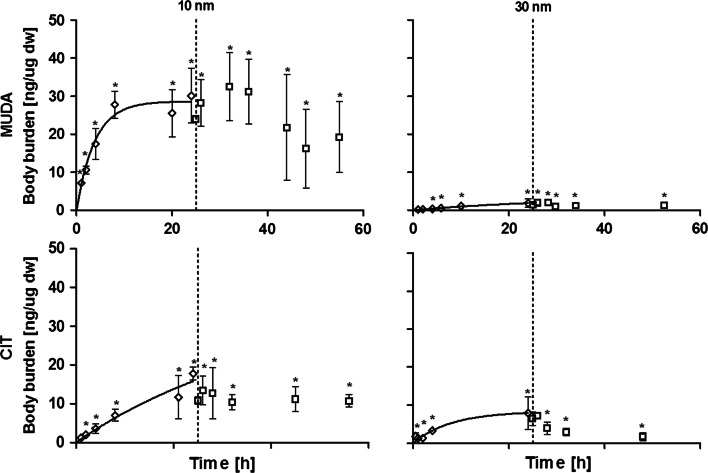
24 h of uptake (*diamonds*) and depuration (*squares*) in neonate *D. magna* during exposure to 0.5 mg Au/L in the uptake phase. The different size and stabilizing agent of the nanoparticles is indicated by the matrix (*MUDA* mercaptoundecanoic acid). Points denoted *asterisk* are statistical significantly different from the control (p < 0.05)

The uptake of Au NP in *D. magna* was assessed by exposing neonates to Au NP for 24 h. For all concentrations and figures reported the respective background concentration in non-exposed control animals was subtracted (0.1 ± 0.03 ng Au/µg dw organism, n = 9). This value was determined as the detection limit using the procedure described in the section “*Chemical analysis*”. Preliminary tests with an uptake period longer than 24 h (48 and 72 h) showed that the body burden in *D. magna*, independent of stabilizing agent or size of Au NP, was not statistically significant different from that of animals exposed for 24 h (p < 0.05) (Fig. S2) thus only data for 24 h was shown here. Similarly, it was found that the aqueous concentration did not show statistically significant changes after 24 h of exposure (Fig. S3). Results of tests with 10 nm MUDA Au NP showed a rapid increase in animal body burden during the first 8 h of the test reaching 27.8 ± 3.6 ng Au/µg dw organism (Fig. 2). After 8 h the uptake stabilized reaching a body burden of 30.1 ± 7.2 ng Au/animal after 24 h. After 24 h of exposure the animals transferred to clean Elendt M7 media showed a decrease in body burden to 24 ± 0.9 ng Au/animal within the first hour of depuration (Fig. 2). From 8 to 24 h of depuration the body burden decreased further to 16.1 ± 10.3 ng Au/animal. Table 3 summarizes the modelled uptake and depuration rates as well as the residual animal body burden after 24 h of depuration. It should be noted that after 24 h of depuration a residual amount of 16.1 ± 10.3 ng Au/µg dw organism of approximately two orders of magnitude higher than the measured background was still present in the animals (Fig. 2).

**Table 3:** Nominal size of particles and stabilizing agent along with modelled uptake and depuration rates, with corresponding R^2^ and the remaining residual body burden of Au at the end of a 24 h depuration period in clean Elendt M7 media

Nominal size (nm)	Stabilizing agent	Uptake rate^a^ (L kg^−1^ dw h^−1^)	Depuration rate (h^−1^)	R^2^	Residual mass (ng Au/µg dw organism)
10	MUDA^b^	4,112–27,720	0.26 (0.15; 0.37)	0.81	16.1 ± 10.3
30	MUDA^b^	35–306	0.03 (0; 0.11)	0.68	1.2 ± 0.76
10	Citrate	339–2,911	0.02 (0; 0.09)	0.84	11.2 ± 3.2
30	Citrate	409–2,275	0.10 (0; 0.25)	0.65	1.7 ± 1.0

The values in the parentheses denote the 95 % confidence interval with upper and lower boundary

^a^The range for the uptake rates were derived from Eq. 1 with the initial water phase concentration (lowest value) and the final water phase concentration (highest value) as input parameters. This was done to accommodate for changes in water concentration during the course of the experiment

^b^Mercaptoundecanoic acid

The test performed with 30 nm MUDA Au NP showed a linear trend of uptake throughout the first 24 h of testing reaching a body burden of 1.83 ± 1.1 ng Au/µg dw organism (Fig. 2). In the depuration phase a general trend of decreasing body burden towards 8 h and flattening towards 28 h was observed (Fig. 2). However, none of the replicates measured were found to be statistically different form each other (p < 0.05) The residual body burden at the end of the depuration study (Table 3) was approximately one order of magnitude higher than the background concentration in non-exposed animals.

Tests with 10 nm CIT Au NP showed an increase in animal body burden up until 24 h of uptake reaching 17.8 ± 1.7 ng Au/µg dw organism (Fig. 2). After transfer to clean medium, a statistically significant decrease in animal body burdens were observed from 0 to 1 h reaching 10.8 ± 0.9 ng Au/µg dw organism. The residual animal body burden reached after 21 h of depuration was 11.2 ± 3.2 ng Au/µg dw organism which is approximately two orders of magnitude higher than the background concentration of Au in control animals.

Results from uptake and depurations studies for 30 nm CIT Au NP are shown in Fig. 2 and Table 3. The data sets from 0.5 to 2 h uptake showed no statistical difference compared to the control but was above the quantification limit of the ICP-OES (0.7 µg Au/L). The data set for 4 h uptake was found to be statistically different from the control with a body burden of 3.3 ± 0.7 ng Au/µg dw organism. After 24 h the body burden had increased to 8.0 ± 4.3 ng Au/µg dw organism. As shown in Fig. 2 the animal body burden decreased to 7.2 ± 0.4 ng Au/µg dw organism within the first hour of the depuration period. From 2 to 4 h a decrease to 3.9 ± 1.7 ng Au/µg dw organism was observed. From 4 to 24 h a trend of decreasing body burden was observed. The residual body burden reached after 24 h of depuration was 1.7 ± 1.0 ng Au/µg dw organism (Table 3) which is, approximately one order of magnitude higher than the measured background concentration in non-exposed control animals.

### Influence of feeding on uptake and depuration of Au NP in *D. magna*

**Fig. 3:**
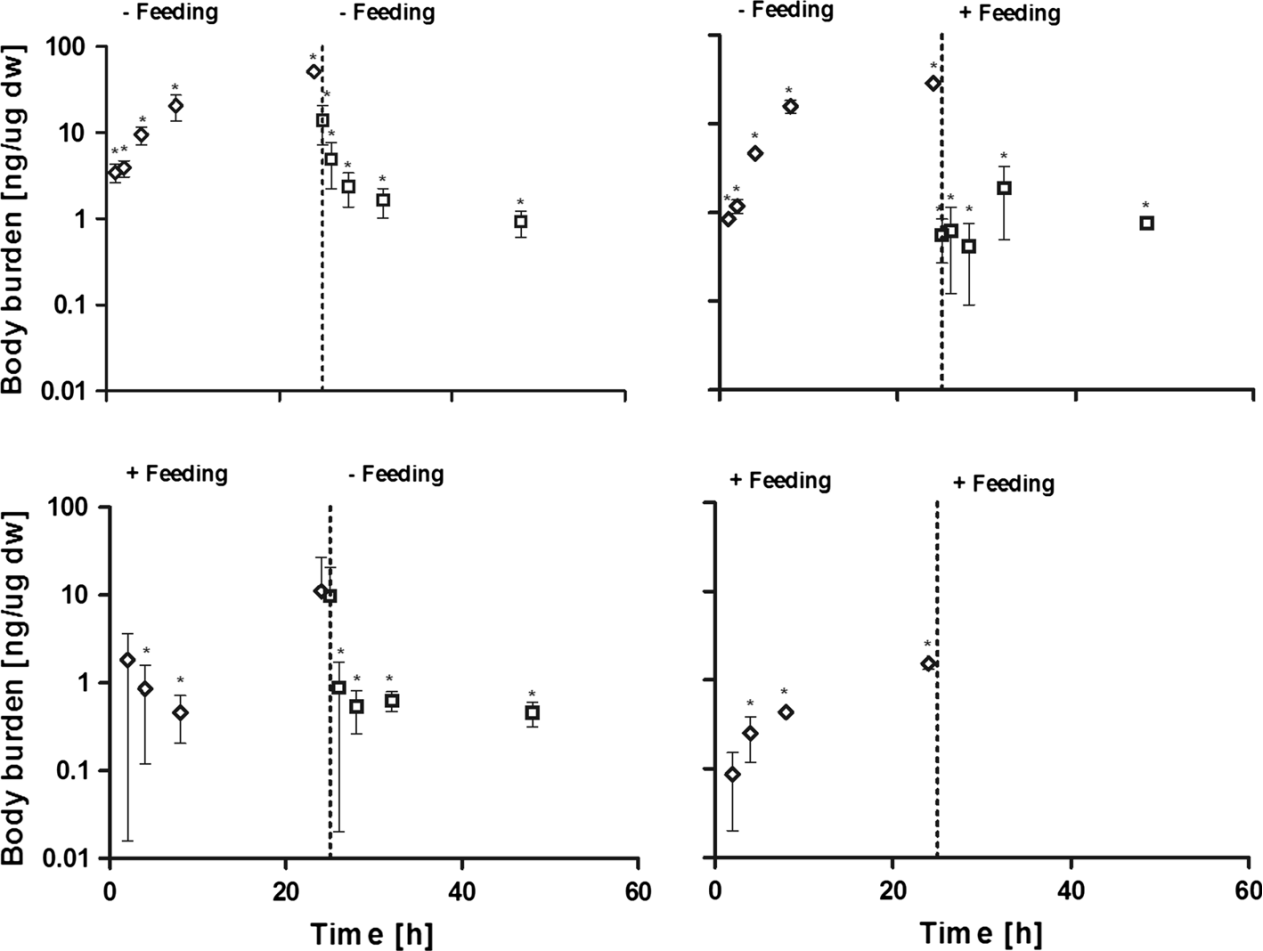
24 h of uptake (*diamonds*) and depuration (*squares*) during exposure to 0.4 mg Au/L with and without food during uptake and depuration using 10 nm CIT Au NP for nanoparticle exposure in the uptake phase. For test with feeding during uptake and depuration all values in the depuration phase was below the detection limit. Points denoted *asterisk* are statistical significantly different from the control (p < 0.05)

The results of experiments carried out to study the influence of feeding during the uptake and depuration of 10 nm CIT Au NP (0.4 mg Au/L) are shown in Fig. 3. For all feeding studies steady body burdens were assumed to be reached after 24 h in accordance with results shown in Figs. 2, 3 and preliminary test results (Fig. S3). Without addition of food in both the uptake and depuration phases, a fast uptake was observed during the first 4 h (Fig. 3). After 24 h of exposure the body burden was 51.3 ± 4.3 ng Au/µg dw organism. After transfer to clean medium a rapid depuration was observed during the first hours (Fig. 3) and levelling off after 8 h. A residual body burden (0.9 ± 0.3 ng Au/animal) of approximately one order of magnitude higher than that of the background concentration of non-exposed control animals was observed after 24 h of depuration.

Tests carried out with no feeding during the uptake phase and feeding during the depuration phase is shown in Fig. 3. An increase in body burden was observed during the first 8 h of the uptake phase and levelled off towards 24 h. The body burden reached after 24 h of uptake was 28.7 ± 4.0 ng Au/µg dw organism. In the depuration phase a rapid decrease in body burden was observed within the first hour after the transfer of animals to clean medium. The data obtained at 2–24 h of depuration showed no statistical difference in the animals’ content of gold compared to that found after 1 h. The residual body burden after 24 h (0.8 ± 0.06 ng Au/µg dw organism) was approximately one order of magnitude higher than that of the measured background concentration.

For the test with feeding during uptake phase and no feeding during depuration the results are shown in Fig. 3. As it was the case for the experiment without feeding during uptake and feeding during depuration, a rapid increase was observed through the first 4 h. The body burden reached after 24 h of uptake was 11.0 ± 15.9 ng Au/µg dw organism. A rapid decrease in animals’ body burdens was observed within the first 2 h after the transfer to clean medium. A residual body burden (0.46 ± 0.14 ng Au/µg dw organism) approximately 1 order of magnitude higher than the background concentration was observed after 24 h of depuration.

Test results for uptake with feeding during both uptake phase and depuration phase is shown in Fig. 3. Even though a rapid increase in body burden was observed during the first 4 h it should be observed that the levels are about a factor of 10 lower than levels observed without feeding (Fig. 3) resulting in a body burden of 1.4 ± 0.2 ng Au/µg dw organism after 24 h. When transferred to clean media the content of gold in the animals was under the detection limit of the ICP-OES already after 1 h.

## Discussion

It is generally assumed that the size exclusion for particle intake by filtration in *D. magna* is in the range 0.4–40 µm. In a study by Lee and Ranville (2012) the size of Au NP used were found to increase from the nominal 20 nm to >1.5 µm after 24 h in hard water, i.e. to sizes where the Au NP might actively be taken up during filtration. Our study confirms that this is the case also for particle sizes below 600 nm as evidenced from the sizes reported in Table 1 and the experiments carried out with dead animals. In the experiments carried out with dead animals no significant uptake was seen supporting the fact that active uptake is the key mechanism for Au NP uptake in *D. magna*. The mass balance of our test system revealed that a substantial amount (38 ± 2.4 % of the mass) of the Au NP added was recovered in *D. magna* after 24 h of exposure. Correspondingly, in the 48 h exposure study of *D. magna* to Au NP, Lee and Ranville (2012) also found a very high (91.2 ± 8.7 %) depletion of Au from an aqueous suspension.

From this it is evident that considerable amounts of added Au NP, dependent on size and agglomeration pattern, is taken up and removed from the water column by *D. magna*.

While the loss of compound due to sorption may not be different from what would be encountered for “conventional” chemicals with low water solubility, the active uptake of particles as well as the possible agglomeration and sedimentation (Unrine et al. 2012; Liu et al. 2012; Tejamaya et al. 2012) highlights that the depletion of nanoparticles from the water column should be accounted for when data from this type of test setup are evaluated. From Fig. 2, a general increase in body burdens with time is observed until steady levels are reached for all Au NP tested. Since the concentration in the beaker is not constant thus assumptions for estimating bioconcentration factors will be invalid even though a plateau is reached. With lower tested concentrations depletion of ENP from the water column could be an issue especially when testing with organisms known to filter large amount of water e.g. mussels or cladocerans. If stripping of ENP from the water column would occur, the idea of diffusion driven transport and chemical equilibrium between the organism and the surroundings would be invalid since the concentration in the media is altered due to active removal of particles into the test organism as indicated from the above studies on mass balance.

A slow depuration of 10 and 30 nm MUDA stabilized Au NP was observed during the first 6 h after transfer to clean media (Fig. 2). Conversely, 10 and 30 nm CIT stabilized Au NP shows a rapid depuration during the first hours after transfer to clean media (Fig. 2). In the literature values varying from 2 to 55 min was found for the gut retention time in *Daphnia* spp. (Bond 1973; Bourne 1959; Rigler 1961; Schindler 1968; McMahon 1970, Gliwicz 1986; Cauchie et al. 2000). Consequently, the depuration of Au NP observed could be a matter of purging of the gut. However, as observed from Fig. 2 there is a substantial residual body burden remaining in the gut of *D. magna* even after the 24 h of depuration, especially for the 10 nm Au NP (Table 3). Gophen and Gold (1981) suggested that *Daphnia* spp. could preserve food in the gut section during starvation. The animals used in our study were not fed during the 48 h of testing and therefore it is likely that the test organism would retain some of their gut content. Figure 2 (squares) shows that ingested Au NP are depurated, possibly through fecal pellets to the test media. However, when no food is present the Au NP may not be bound in fecal pellets and may re-enter the water column and be available for uptake. The behavioural traits of *D. magna* to scavenge bottom sediments(the bottom of the glassware in this type of test setup) searching for food sources, may imply that excreted Au NP may still be available for uptake. In our test setup the role of fecal pellets in Au NP uptake could not be evaluated, but since other studies have found significant amounts of ENP in feces of test organisms e.g. in mussels by Montes et al. (2012), the influence hereof on uptake of ENP should be studied further.

A lower uptake of MUDA 30 nm Au NP in terms of mass was observed through the whole uptake period compared to the other Au NP (Fig. 2). The differences in stabilizing agents and sizes may have resulted in different agglomeration behaviour in the media rendering differences in bioavailability of the tested Au NP. Liu et al. (2012) used Au NP of same type and same batch as those applied in the present study and found that a combination of stabilizing agent and particle size affected the agglomeration kinetics. Thus, the results for uptake and depuration in the present study were found to be in agreement with behaviour of Au NP described by Liu et al. (2012).

The modelled uptake rates for CIT 10 nm and CIT 30 were within the same order of magnitude (Table 3). While the depuration rate for MUDA 10 nm and MUDA 30 Au NP showed a respectively faster and slower release of ingested particles compared to the CIT stabilized Au NP. These findings suggest that stabilizing agents and initial particle sizes is important for determining the uptake and depuration behavior (Table 3). Results from Liu et al. (2012) suggested that agglomeration behaviour of Au NP is more dependent on their coating and stabilizing agent compared to core composition and particle size. Similarly, it was shown in this study that differences in stabilizing agent altered the agglomeration pattern (Table 1) but also that changes occurred as a function of time. Handy et al. (2012a) emphasized the importance of maintaining control of the test setup in terms of e.g. test media and establishing concentrations during testing of ENP. The presented test setup offers the advantage that it uses a relatively short incubation period (in total 48 h). Hereby the possibilities for controlling and characterizing ENP exposure during incubation (for an extended discussion on test setup considerations using ENM see the review by Handy et al. (2012b)). However, it should be noted that complete depurations of Au NP from the animals were not obtained within the 24 h depuration period applied in the present study. Consequently, additional purging of the gut could be necessary to distinguish between Au NP situated in the gut and in other tissues (Gillis et al. 2005). Feeding often facilitates purging or clearing of the gut and the results shown in Fig. 3 also demonstrate that the addition of food affects the outcome of the tests. Both with and without the addition of algae, a rapid uptake during the first 2 h of the test was observed (Fig. 3). However, the body burden after 24 h differed depending on the presence or absence of food during uptake (Fig. 3). The body burden after 24 h reached 8.8 ± 12.7 ng Au/μg dw organism when food was present compared to 26.1 ± 2.2 ng Au/μg dw organism without food (Fig. 3). It is possible that sorption of Au NP to algae followed by ingestion obscures the clear uptake patterns generally seen in the absence of food in the uptake period. The indication of lower body burdens due to addition of food could also be caused by increased purging, as discussed previously.

Consequently, it is clear that the presence of food adds another level of complexity to the test setup and increase the difficulty to achieve controlled conditions. However, as presented in the above study the highest body burden were seen when no feeding was done, and thus a worst-case scenario may be achieved when addition or presence of food is avoided. As addition of food to a larger extend resemble the processes that will occur in the environment a test setup with feeding will create a better understanding for what would happen in the event of ENM being released. An important aspect is that the lack of food seems to overestimate the uptake of ENM.

## Conclusion

This study showed the feasibility of a short-term study using the invertebrate *D. magna* for assessing the uptake and depuration of Au NP as models for non-reactive ENP. The findings underlines that the assumptions behind the traditional way of quantifying bioconcentration are not fulfilled when ENPs are studied since steady state and equilibrium chemistry do not apply to colloidal suspensions undergoing dynamic changes during the incubation. Based on mass balance measurements during the 24 h exposure period it was found that five neonate *D. magna* can take up more than one-third of the added 0.5 mg Au/L in 25 mL suspensions of 10 nm CIT stabilized Au NP. No sorption of Au NP to exterior surface of the test animals was found for the tested types of Au NP. A fast initial uptake in *D. magna* neonates was observed independent of size and stabilizing agent. However, the results indicate that stabilizing agent affected the depuration rate, though there was no trend in size. The residual concentration in animals after 24 h of depuration seemed to be more related to particle size than particle stabilizing agent as the 10 nm Au NP were found in higher amounts than the 30 nm Au NP regardless of stabilizing agent. The residual body burdens of 10 nm Au NP were about two orders of magnitude higher than that of the control and one order of magnitude higher than that of the 30 nm Au NP. While it was found that feeding did not significantly affect the uptake of 10 nm CIT Au NP, faster depuration was measured when animals were fed. This finding may have implications for long term studies of ENP in *D. magna* where feeding is necessary.

## Electronic supplementary material

Below is the link to the electronic supplementary material.

Fig. S1 IR spectra of CIT Au NP and MUDA Au NP (left) and XPS after ligand exchange in aqueous solution in MUDA Au NP (right) (TIFF 2104 kb)

Fig. S2 Aqueous phase concentration of 0.4 mg Au/L after 24h, 48h and 72h in the presence of *D. magna*. Concentrations were measured at time 0 and calculated as percentage of initial (time 0) (TIFF 245 kb)

Fig. S3 Body burden of Au NP with different stabilizing agents in *D*. *magna* after exposure to 0.4 mg Au/L for 24h, 48h and 72h (TIFF 223 kb)

Table S1 Conditions for reference test and EC50-value for 48 hours using potassium dichromate (DOCX 15 kb)

Table S2 Nominal size of particles and stabilizing agent along with modelled uptake (Start) and uptake (Final) rates, with corresponding R^2^. The values in the parentheses denote the 95% confidence interval with upper and lower boundary (DOCX 17 kb)
