# Supplementary figures and images for: Uptake and depuration of gold nanoparticles in Daphnia magna
Source: Ecotoxicology. 2014 May 27;23(7):1172–83. doi: 10.1007/s10646-014-1259-x (PMC4131140; doi:10.1007/s10646-014-1259-x)

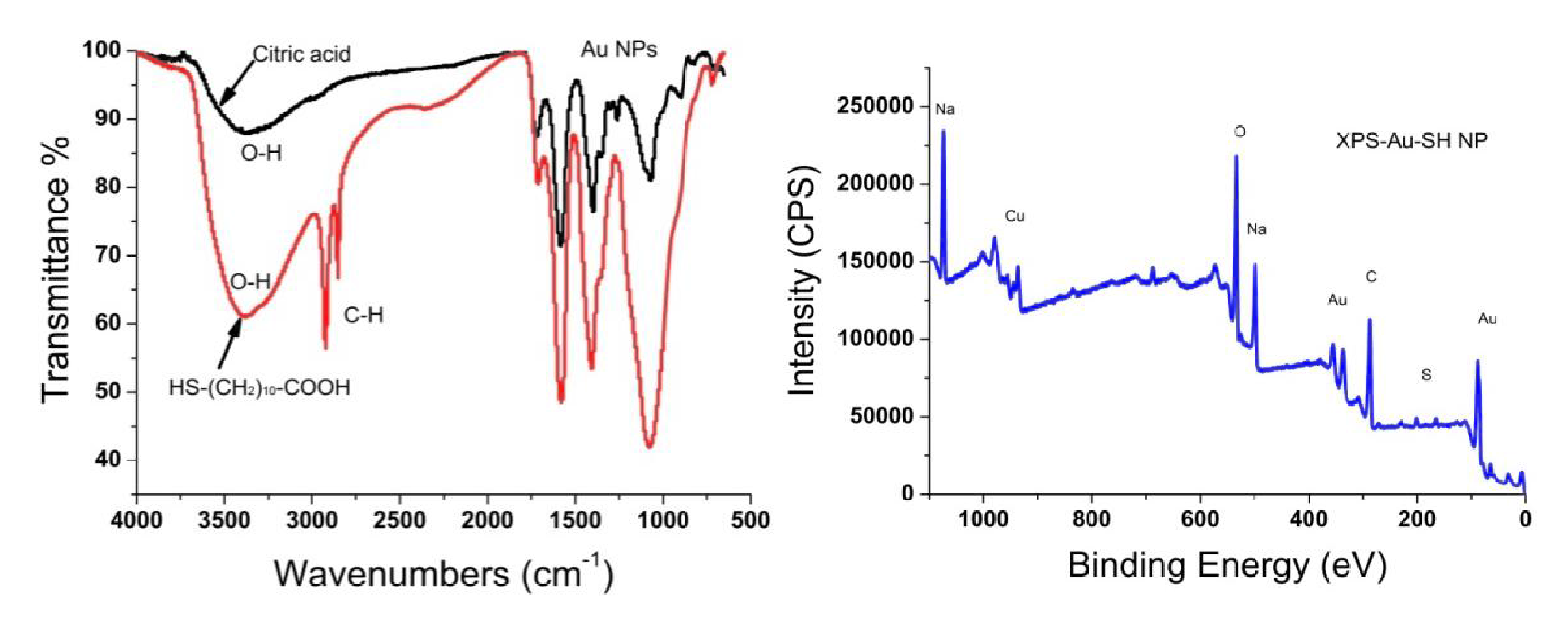

Supplement: Supplementary file 1 — Fig. S1 IR spectra of CIT Au NP and MUDA Au NP (left) and XPS after ligand exchange in aqueous solution in MUDA Au NP (right) (TIFF 2104 kb) [file 10646_2014_1259_MOESM1_ESM.tif]

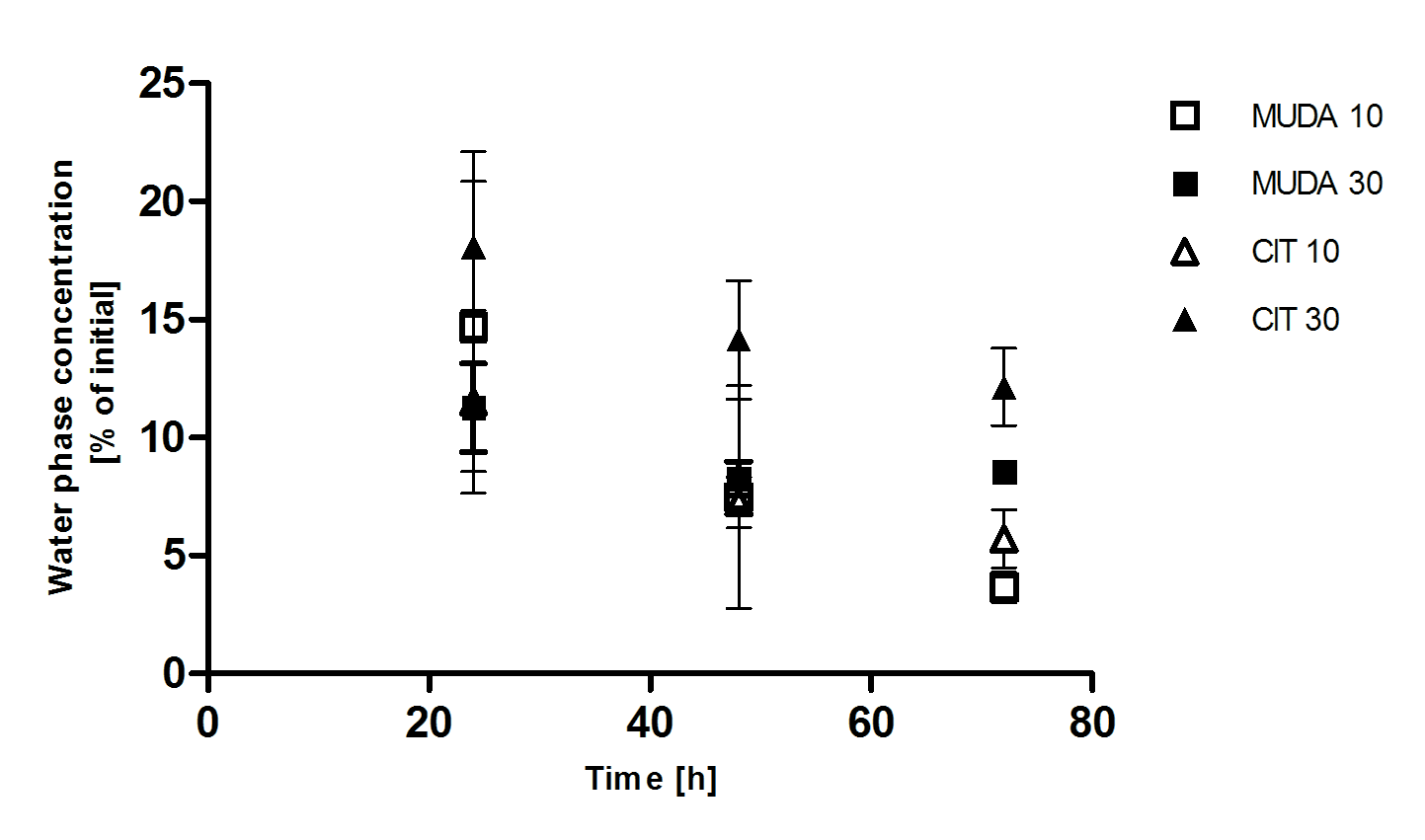

Supplement: Supplementary file 2 — Fig. S2 Aqueous phase concentration of 0.4 mg Au/L after 24h, 48h and 72h in the presence of D. magna. Concentrations were measured at time 0 and calculated as percentage of initial (time 0) (TIFF 245 kb) [file 10646_2014_1259_MOESM2_ESM.tif]

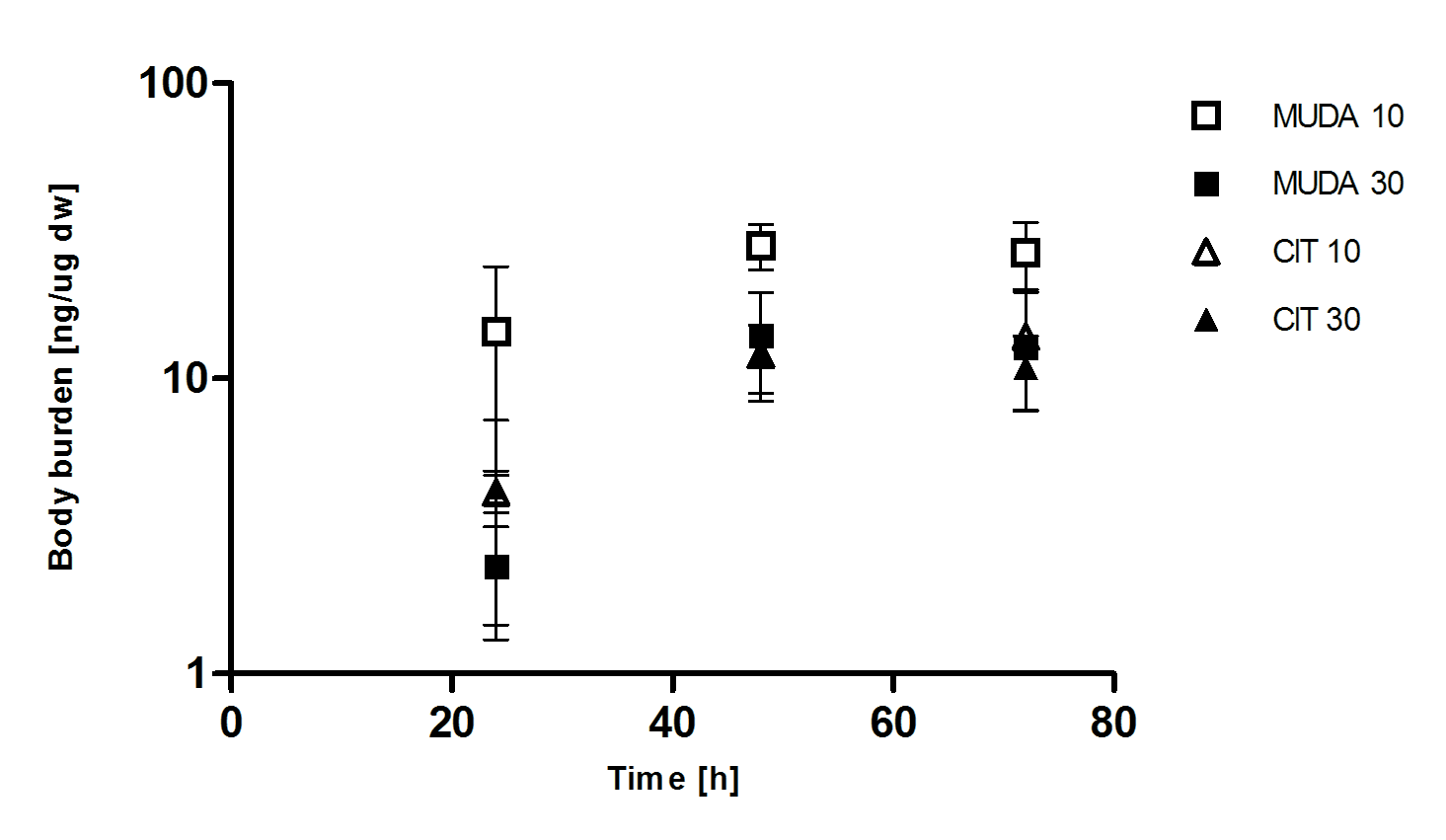

Supplement: Supplementary file 3 — Fig. S3 Body burden of Au NP with different stabilizing agents in D. magna after exposure to 0.4 mg Au/L for 24h, 48h and 72h (TIFF 223 kb) [file 10646_2014_1259_MOESM3_ESM.tif]
