# Supplementary material for: Uptake and depuration of gold nanoparticles in Daphnia magna
Source: Ecotoxicology. 2014 May 27;23(7):1172–83. doi: 10.1007/s10646-014-1259-x (PMC4131140; doi:10.1007/s10646-014-1259-x)
Supplement: Supplementary file 4 — Table S1 Conditions for reference test and EC50-value for 48 hours using potassium dichromate (DOCX 15 kb) [file 10646_2014_1259_MOESM4_ESM.docx]

| Parameter | Oxygen  [mg/L] | pH | EC_50. 48h_ (K_2_Cr_2_O_7_) |
| --- | --- | --- | --- |
| Start | 8.8 | 7.6 | N/A |
| End | 8.7 | 7.4 | 0,91 |
