# Supplementary material for: Uptake and depuration of gold nanoparticles in Daphnia magna
Source: Ecotoxicology. 2014 May 27;23(7):1172–83. doi: 10.1007/s10646-014-1259-x (PMC4131140; doi:10.1007/s10646-014-1259-x)
Supplement: Supplementary file 5 — Table S2 Nominal size of particles and stabilizing agent along with modelled uptake (Start) and uptake (Final) rates, with corresponding R2. The values in the parentheses denote the 95% confidence interval with upper and lower boundary (DOCX 17 kb) [file 10646_2014_1259_MOESM5_ESM.docx]

| Nominal size  [nm] | Stabilizing agent | Uptake rate (Start)^a^  [L kg^-1^ dw h^-1^] | Uptake rate (Final)^b^  [L kg^-1^ dw h^-1^] | R^2^ |
| --- | --- | --- | --- | --- |
| 10 | MUDA* | 4112 (2691; 5534) | 27720 (18140; 37300) | 0.81 |
| 30 | MUDA* | 35 (8; 61) | 306 (73; 539) | 0.68 |
| 10 | Citrate | 339 (129; 548) | 2911 (1113; 4709) | 0.84 |
| 30 | Citrate | 409 (88; 729) | 2275 (492; 4059) | 0.65 |

^a^ Uptake rates were modelled with the initial water phase concentration (t=0h). ^b^ Uptake rates were modelled were modelled with the final water phase concentration (t=24h). *mercaptoundecanoic acid
